# Association of Race With Urine Toxicology Testing Among Pregnant Patients During Labor and Delivery

**DOI:** 10.1001/jamahealthforum.2023.0441

**Published:** 2023-04-14

**Authors:** Marian Jarlenski, Jay Shroff, Mishka Terplan, Sarah C. M. Roberts, Brittany Brown-Podgorski, Elizabeth E. Krans

**Affiliations:** 1 Department of Health Policy and Management, University of Pittsburgh School of Public Health, Pittsburgh, Pennsylvania; 2 Friends Research Institute, Baltimore, Maryland; 3 Department of Obstetrics, Gynecology, and Reproductive Sciences, University of California, San Francisco; 4 Department of Obstetrics, Gynecology & Reproductive Sciences, University of Pittsburgh School of Medicine, Pittsburgh, Pennsylvania; 5 Magee-Womens Research Institute, Pittsburgh, Pennsylvania

## Abstract

This cohort study assesses the association of race with receipt of urine toxicology testing and a positive test result among pregnant patients admitted to the hospital for delivery.

## Introduction

An estimated 16% of pregnant persons in the US use alcohol (10%) or an illicit substance (6%, including cannabis).^1^ Urine toxicology testing (UTT) is often performed at the time of labor and delivery for pregnant patients to evaluate substance use.^2,3^ We sought to elucidate associations between race and receipt of UTT and a positive test result among pregnant patients admitted to the hospital for delivery.

## Methods

This cohort study followed the STROBE reporting guideline. Data were extracted from electronic medical records (EMRs) of patients with a live or stillbirth delivery between March 2018 and June 2021 in a large health care system in Pennsylvania. The study was approved by the University of Pittsburgh institutional review board. Informed consent was waived because the research constituted minimal risk. All patients presenting for delivery were verbally screened for substance use using questions adapted from the National Institute on Drug Abuse Quick Screen.^4^ Policy specified UTT would be performed for those with a positive screen result, history of substance use in the year prior to delivery, few prenatal visits, or abruption or stillbirth without a clear medical explanation.

We studied 2 binary outcomes: the receipt of UTT (point of care presumptive testing) and a positive test result at delivery. The primary variable of interest, patient race, was conceptualized as a social construct that could manifest in biased or discriminatory delivery of health care. Self-reported race was categorized as Black, White, and other (Alaska Native, American Indian, Chinese, Filipino, Guam/Chamorro Hawaiian, Indian, Japanese, Korean, Other Asian/Pacific Islander, Samoan, and Vietnamese). Substance use history was defined as having a diagnosis of an alcohol, cannabis, opioid, or stimulant use or disorder during pregnancy in the EMR within 1 year prior through delivery. A positive UTT result was defined as at least 1 positive result of a test component, including amphetamines, barbiturates, benzodiazepines, buprenorphine, cocaine, cannabis, methadone, opiates, or phencyclidine. We used multivariable logistic regression models including race and substance use history, adjusting for age, Hispanic or Latina/x ethnicity, marital status, parity, tobacco use, prenatal visit utilization, stillbirth, and placental abruption. We derived mean predicted probabilities of outcomes by race and substance use history.^5^ Analyses were conducted using Stata, version 17.

## Results

Among 37 860 patients (100% female; mean [SD] age, 29.8 [5.5] years), 16% Black, 76% were White, and 8% were other race (Table). Overall, 11% had a history of substance use; opioid use was more common among White patients (40% of all substance use), whereas cannabis use was most common among Black patients (86% of all substance use). The mean predicted probability of having a UTT at delivery was highest among Black patients compared with White patients and other racial groups regardless of history of substance use (Figure). For Black patients without a history of substance use, the mean predicted probability of receiving a UTT at delivery was 6.9% (95% CI, 6.4%-7.4%) vs 4.7% (95% CI, 4.4%-4.9%) among White patients. Among Black patients with a history of substance use, the mean predicted probability of receiving a UTT at delivery was 76.4% (95% CI, 74.8%-78.0%) vs 68.7% (95% CI, 67.3%-70.1%) among White patients. In contrast, among those with a history of substance use, the mean predicted probability of having a positive test result was 66.7% (95% CI, 64.8%-68.7%) among White patients and 58.3% (95% CI, 55.5%-61.1%) among Black patients.

**Table.** Descriptive Characteristics of Patients Delivering in the Healthcare System Overall and Stratified by Race Categories

Characteristic	Patients, No. (%)			
	Overall (n = 37 860)	Black (n = 6061)	White (n = 28 797)	Other (n = 3002)^a^
Age at delivery, y				
<19	545 (1)	196 (3)	328 (1)	21 (1)
19-25	8223 (22)	2189 (36)	5601 (19)	433 (14)
26-34	21 423 (57)	2862 (47)	16 765 (58)	1796 (60)
≥35	7669 (20)	814 (13)	6103 (21)	752 (25)
Hispanic or Latina/x ethnicity	874 (2)	67 (1)	611 (2)	196 (7)
Parity^b^	31 397 (83)	5079 (84)	23 878 (83)	2439 (81)
Not married^c^	19 104 (50)	5234 (86)	12 984 (45)	886 (30)
Substance use history in 12 mo before delivery				
Any^d^	4100 (11)	1288 (21)	2723 (9)	89 (3)
Cannabis	2488 (7)	1112 (19)	1337 (5)	39 (1)
Opioids	1213 (3)	97 (2)	1095 (4)	21 (1)
Stimulants	461 (1)	106 (2)	347 (1)	8 (<1)
Alcohol	187 (<1)	68 (1)	117 (<1)	2 (<1)
Tobacco^d^	5767 (15)	1427 (24)	4229 (15)	111 (4)
Other urine toxicology criteria^e^				
Few prenatal care visits^f^	11 115 (29)	1878 (31)	8287 (29)	950 (32)
Stillbirth	223 (<1)	56 (1)	155 (1)	12 (<1)
Placental abruption	664 (2)	150 (2)	461 (2)	53 (2)
UTT administered at delivery	4636 (12)	1526 (25)	2973 (10)	137 (5)
Positive UTT result	2199 (47)	610 (40)	1547 (52)	42 (31)

Abbreviation: UTT, urine toxicology testing.

^a^ Other races include Alaska Native, American Indian, Chinese, Filipino, Guam/Chamorro Hawaiian, Indian, Japanese, Korean, Other Asian/Pacific Islander, Samoan, and Vietnamese.

^b^ Indicates having any previous birth.

^c^ At any time in pregnancy.

^d^ Identified in medical record at any time during the year prior to delivery through the delivery.

^e^ In the health care system, these additional events would trigger a UTT in the absence of a history of substance use.

^f^ Less than 4 prenatal care visits, or prenatal care initiation after 27 weeks gestation.

**Figure.**
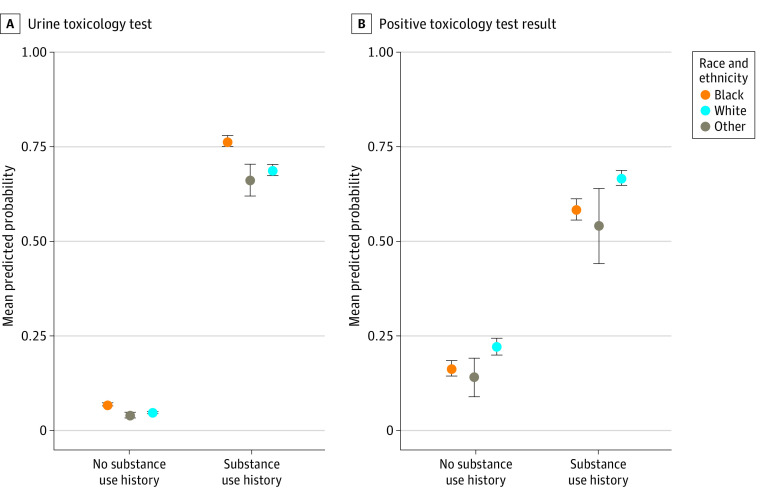
Mean Predicted Probability of Having a Urine Toxicology Test or Having a Positive Test Result at Delivery Results are from logistic regression models controlling for age, Hispanic or Latina/x ethnicity, marital status, parity, tobacco use, prenatal visit utilization, stillbirth, and placental abruption. Other race includes Alaska Native, American Indian, Chinese, Filipino, Guam/Chamorro Hawaiian, Indian, Japanese, Korean, Other Asian/Pacific Islander, Samoan, and Vietnamese. In the sample, 4100 patients had a history of substance use, and 33 760 had no history of substance use; 4636 had a urine toxicology test, and 2199 had any positive test result at labor and delivery. Error bars indicate 95% CIs.

## Discussion

In this cohort study, Black patients, regardless of history of substance use, had a greater probability of receiving a UTT at delivery compared with White patients and other racial groups. However, Black patients did not have a higher probability of a positive test result than other racial groups. Limitations of the study include a lack of a sufficient sample size to investigate other racial and ethnic minoritized groups, such as Alaska Native and American Indian patients, and that data were from a single geographical area and may not generalize nationally. To address racial biases, health care systems should examine drug testing practices and adhere to evidence-based practices.

## Notes

Supplement.
Data Sharing Statement
