# Supplementary material for: Association of Race With Urine Toxicology Testing Among Pregnant Patients During Labor and Delivery
Source: JAMA Health Forum. 2023 Apr 14;4(4):e230441. doi: 10.1001/jamahealthforum.2023.0441 (PMC10105305; doi:10.1001/jamahealthforum.2023.0441)
Supplement: Supplement. — Data Sharing Statement [file jamahealthforum-e230441-s001.pdf]

Data Sharing Statement

Jarlenski. Association of Race With Urine Toxicology Testing Among Pregnant Patients During Labor and Delivery. *JAMA Health Forum*. Published April 14, 2023.
doi:10.1001/jamahealthforum.2023.0441

Data
Data available: No

Additional Information
Explanation for why data not available: The data are from electronic medical records and are not authorized to be shared under the data use agreement.
